# Malleability and fluidity of time perception

**DOI:** 10.1038/s41598-024-62189-7

**Published:** 2024-05-29

**Authors:** Hirohito M. Kondo, Elena Gheorghiu, Ana P. Pinheiro

**Affiliations:** 1 School of Psychology, Chukyo University, 101-2 Yagoto Honmachi, Showa, Nagoya, Aichi 466-8666 Japan; 2 Department of Psychology, Faculty of Natural Sciences, University of Stirling, Stirling, UK; 3 Faculty of Psychology, University of Lisbon, Lisbon, Portugal

**Keywords:** Human behaviour, Perception

## Abstract

Time perception is inherently subjective and malleable. We experience a wide range of time scales, from less than a second to decades. In addition, our perception of time can be affected by our attentional and emotional states. Previous psychological and neuroimaging studies have used several paradigms and methods to probe factors that influence time perception. Considering these factors facilitates approaches to improve time management and to enhance sensory experiences. This Collection of time perception studies includes reports that focus on stimulus property, physiological state, cross-modal interaction, attention, learning, age, and environment. These findings help to illuminate the complex mechanisms of time perception.

Humans have no absolute sense of time. Time perception is fundamentally subjective and depends on one's experiences and circumstances. Moments of excitement and joy can seem dizzyingly faster, whereas moments of boredom and stress can feel interminable^1^, illustrating how attentional and emotional states affect time perception. Also, time perception has critical effects on many cognitive abilities and motor skills. For instance, we can play the piano with quick movements. Accurate temporal and rhythmic performance are important not only for playing music, but also for multisensory perception, language, and motor planning^2,3^. In addition, we may vividly recall memories from years ago. We have a broad range of time scales^4^. These timings appear to be underpinned by different neural mechanisms^5^. The brain navigates and processes time ranges from subsecond to year, highlighting its remarkable adaptability and complexity.

The more often we pay attention to the passage of time, the longer we perceive time to be^6^. Our perception of the passage of time may vary as a function of age and education^7^ or mood state^8^. This is probably consistent with the contextual-change hypothesis that the perceived duration of an event is affected by the number of contextual changes^9^. Extending this idea may explain how different age groups perceive time differently. For boys and girls, holiday adventures are hard to come by. Adults have many routine activities and time seems to pass at an accelerated pace. Relative to adults, children may use heuristic methods for duration estimation^10^. However, it should be noted that feeling the passage of time and estimating duration may employ different mechanisms of time perception^11^.

A simple explanation for the perceived compression and expansion of time is the event-density hypothesis. This postulates that the number of events occurring during a certain period affects perception of time intervals^12^, assuming that the “internal clock” counts at a constant rhythm^13^. Directing attention to salient stimuli or engaging in complex tasks increases internal pulses, i.e., the density of events, resulting in the perception that time is passing quickly^6^. This hypothesis is consistent with the idea that cellular metabolism and the internal clock are intimately interconnected. An early study argued that as body temperature increases, the internal clock seems to advance faster, leading to the perception of shorter durations^14^. Cognitive components, such as working memory and attention, were incorporated into the pulse-generating pacemaker and developed into the scalar expectancy model^15^ and the attentional-gate model^16^.

Time perception depends not only on endogenous factors, such as attentional, motivational, and physiological levels^17,18^, but also on exogenous factors, such as speed of motion, stimulus complexity^19^, salience of visual stimulus features^20^, and spatial, temporal, social context^21^ or environment^22^. Previous studies have frequently employed experimental paradigms such as temporal order or duration judgements to assess time perception of short intervals. In such paradigms, a novel or “oddball” stimulus is perceived as longer in duration than repeated or “standard” stimuli^23^. The first visual stimulus in a train appears to be perceived as longer than successive stimuli^24^. However, such a phenomenon does not occur in relation to auditory stimuli. There is a consensus that timing of subsecond intervals is supported by distributed sensory-specific mechanisms^25,26^. An event-related potential (ERP) study demonstrated that people with normal hearing, but not deaf individuals, show a strong ERP response to visual stimuli in temporal areas during a time-bisection task, whereas the same response is not elicited during a space-bisection task^27^ (see also a study of developmental viewpoint^28^). Gamma-aminobutyric acid (GABA) levels in the human visual cortex measured using magnetic resonance spectroscopy appear to correlate with perceived durations of visual intervals, suggesting that the GABAergic system contributes to individual differences in time perception^29^. However, time perception studies in this Collection have found that learning of temporal interval discrimination transfers between auditory and visual modalities^30^ and that cross-modal correspondence between auditory pitch and visual elevation selectively affects temporal recalibration^31^.

There is no single sensory organ responsible for time perception. Different brain regions are involved in temporal processing depending on time scales. Subsecond time intervals are mainly processed in the cerebellum^32^, whereas temporal processing in the range of seconds and minutes is supported by the prefrontal cortex and striatum^33,34^. In addition, time perception is impaired in disorders of the precuneus/posterior cingulate gyrus^35^ and supramarginal gyrus^36^. In particular, the precuneus may contribute to our sense of “presentness”, providing the “now” in the passage of time^37,38^.

The advent of digital technology has had an unprecedented impact on time perception. Ubiquitous access to the Internet facilitates instantaneous information retrieval and synchronous communication. A consequence of this persistent connectivity is the potential for information overload, such that the sense of time tends to become ambiguous. The widespread prevalence of social media notifications may contribute to the perceived acceleration of time. However, through flow and meditation states, alternative perceptions of time can be experienced. Specifically, a flow experience is a symbolic phenomenon of time distortion, in which one forgets the passage of time by immersing oneself in a certain activity. People in a flow state often report this state as being “in the zone”^39^. Although there are anecdotal reports of flow experiences by athletes, few studies have captured quantitative aspects of flow states^40^. However, some studies have identified flow states in terms of attentional fluctuations^41,42^. Using such methods, it may be possible to overcome methodological difficulties and to measure altered time perception.

Articles in this Collection show that the interplay of stimulus property, physiological state, attention, age, and environment fundamentally shapes individual temporal experiences. A deep understanding of these factors is undoubtedly crucial to the ongoing field of time perception research.
